# Postoperative intravenously administered iron sucrose versus postoperative orally administered iron to treat post-bariatric abdominoplasty anaemia (ISAPA): the study protocol for a randomised controlled trial

**DOI:** 10.1186/s13063-016-1300-x

**Published:** 2016-04-12

**Authors:** Juan Carlos Montano-Pedroso, Elvio Bueno Garcia, Neil Ferreira Novo, Daniela Francescato Veiga, Lydia Masako Ferreira

**Affiliations:** Division of Plastic Surgery, Graduate Program in Translational Surgery, Federal University of São Paulo, Rua Napoleão de Barros, 725, 4th Floor, Vila Clementino, CEP: 04024-002 São Paulo, SP Brazil

**Keywords:** Anaemia, Iron, Abdominal wall, Plastic surgery, Bariatric surgery, Haemoglobin, Adult, Female

## Abstract

**Background:**

Anaemia and iron deficiency are common complications following post-bariatric abdominoplasty. Given the low oral absorbability of iron resulting from bariatric surgery, it has been hypothesised that postoperative intravenously administered iron supplementation could be used to treat anaemia and to prevent the development of iron deficiency in these patients.

**Methods/Design:**

In this multicentre open-label randomised clinical trial, 56 adult women undergoing post-bariatric anchor-line abdominoplasty will be allocated at a ratio of 1:1 for postoperative supplementation with two intravenously administered applications of 200 mg of iron saccharate or postoperative supplementation with 100 mg of iron polymaltose complex administered orally, twice a day for 8 weeks. The primary outcome is the difference in mean haemoglobin levels between the two groups at eight postoperative weeks. Secondary outcomes evaluated at one, four and eight postoperative weeks include iron profile, reticulocyte count, overall quality of life measured using the Short-Form 36 Health Survey (SF-36) questionnaire, fatigue measured using the Functional Assessment of Chronic Illness Therapy – Fatigue (FACIT-F), adverse effects and postoperative complications.

**Discussion:**

This randomised clinical trial aims to evaluate the haematopoietic effectiveness of intravenously administered iron supplementation in patients undergoing post-bariatric abdominoplasty. A more effective recovery of haemoglobin levels could help improve the patients’ quality of life and could provide an improved haematological status in preparation for the subsequent and frequent plastic surgeries these patients undergo.

**Trial registration:**

Clinicaltrials.gov Identifier: NCT01857011 (8 May 2013), Universal Trial Number U111-1169-6223, Brazilian Clinical Trials Registry (REBEC): RBR-2JGRKQ.

## Background

The increasing prevalence of overweight and obesity in many countries has been described as a global pandemic. The number of overweight and obese individuals has increased from 857 million in 1980 to 2.1 billion worldwide in 2013 [1]. The proportion of adults with body mass indexes (BMIs) above 25 kg/m^2^ has increased between 1980 and 2013 from 28.8 to 36.9 % in men and from 29.8 to 38 % in women [1]. Obesity is associated with increased risk of type 2 diabetes, hypertension, dyslipidaemia, cardiovascular diseases, some types of cancer and mortality [2]. Overweight and obesity were estimated to have caused 3 to 4 million deaths worldwide in 2010, constituting the sixth most important risk factor for disease development worldwide [3].

Treatment options for obesity include different non-surgical treatments and bariatric surgery. Compared to non-surgical treatment, bariatric surgery results in greater weight loss and an increase in the remission rate of type 2 diabetes and metabolic syndrome [2]. However, even though patients undergoing bariatric surgery successfully lose weight, 96 % of patients develop a number of body-contouring deformities caused by excess skin and residual fat deposits [4, 5].

In addition to causing intertrigo and hygiene and mobility difficulties, skin deformities have the potential to affect body image, self-esteem and quality of life [6]. Of the body regions that develop deformities, patients tend to complain the most about the abdomen [7]. After significant weight loss, 75 % of female patients wish to perform body-contouring surgery [5, 8].

However, post-bariatric patients are 60–87 % more susceptible to complications associated with body-contouring surgery compared to non-bariatric patients [9]. Dietary deficiencies are common, with 51.3 % of patients developing iron deficiency and a 36 % prevalence of anaemia [10, 11]. The primary causes of these conditions are reduction in iron intake, gastric acid secretion and contact of food with the iron absorption areas of the duodenal and jejunal segments separated from the gastrointestinal tract as a result of bariatric surgery [12].

During body-contouring surgery, such as post-bariatric abdominoplasty, large areas of subcutaneous tissue and highly vascularised skin are removed and may result in considerable blood loss. Two months after surgery, patients normally still have not returned to preoperative haemoglobin (Hb) levels, and 45 % develop severe iron deficiency [13].

Anaemia is the second most common complication during the postoperative period following post-bariatric abdominoplasty, [7] and these patients have a higher indication for blood transfusion [14]. However, observational studies have found an association between blood transfusion and higher comorbidity and mortality [15–17], and in patients undergoing post-bariatric abdominoplasty, transfusions are associated with a two-fold higher complication rate and longer hospital stays [18].

Moreover, in surgical patients, even low levels of anaemia have been associated with adverse outcomes [19–22]. The recovery of Hb levels is considered an important factor in the postoperative recovery of patients [23], especially in post-bariatric patients, because they are often subjected to additional surgical procedures to treat breast, thigh and arm deformities over relatively short periods of time [24]. In addition, several studies have found that iron deficiency, even in non-anaemic patients, can cause symptoms of fatigue; correcting this deficiency helps improve this symptom [25–27].

Iron supplementation can be administered either orally or parenterally; however, intravenous administration is five-fold more effective in inducing an erythropoietic response after significant blood loss [28]. In addition, orally administered iron can induce side effects such as gastrointestinal symptoms, jeopardising adherence to treatment [29]. Moreover, adverse effects caused by the use of intravenously administered iron, with the exception of high-molecular-weight iron dextran, are rare [30]. Observational studies on orthopaedic surgery [31], digestive tract surgery [32], postpartum haemorrhage [33] and renal transplantation [34, 35] have demonstrated the efficacy of intravenously administered iron in optimising the recovery of Hb levels.

However, a narrative review analysed the results of four randomised controlled trials that evaluated the impact of the use of intravenously administered iron on the perioperative status of cardiac and orthopaedic surgical patients and found that there were no benefits in the recovery of Hb levels or reductions in the number of blood transfusions [36]. In turn, a systematic review evaluated the effects of iron in anaemic patients without chronic kidney disease and observed higher Hb levels with intravenous administration of iron compared to oral administration, but without clinical benefit in terms of reducing blood transfusions or improving quality of life [37]. None of these reviews included patients undergoing post-bariatric surgery. Due to the difficulty in absorbing iron orally and their low levels of stored iron [10], there is the possibility that intravenously administered iron supplementation can result in a better recovery of Hb levels, a decrease in iron deficiency and, consequently, improvements in the patients’ quality of life [13, 38–40].

### Objectives

#### Primary objective

To assess whether iron saccharate supplementation intravenously administered in two 200-mg doses during the perioperative period of post-bariatric anchor-line abdominoplasty results in an increase in Hb levels in the eighth postoperative week (study group) compared to orally administered iron supplementation (control group).

#### Secondary objectives

To investigate whether the use of intravenously administered iron has beneficial effects on other haematological variables, iron profile, transfusion rate, quality of life and clinical outcomes.

## Methods/Design

### Trial design

This is a parallel, superiority, randomised controlled clinical trial with an allocation ratio of 1:1 (Table 1).

**Table 1:** World Health Organisation (WHO) trial registration data set

Primary registry and trial identifying number	NCT01857011
Date of registration in primary registry	8 May 2013
Secondary identifying numbers	U1111-1169-6223, Brazilian Clinical Trials Registry (REBEC): RBR-2JGRKQ
Source of monetary or material support	São Paulo Research Foundation (Fundação de Amparo à Pesquisa do Estado de São Paulo – FAPESP), grant #2014/07710-6
Primary sponsor	Federal University of São Paulo
Secondary sponsor	Instituto de Assistência Médica ao Servidor Público Estadual (IAMSPE)
Contact for public queries	Juan Carlos Montano Pedroso (juancmontano@gmail.com)
Contact for scientific queries	Juan Carlos Montano Pedroso (juancmontano@gmail.com0
Public title	Iron Supplementation for the Treatment of Acute Anaemia After Postbariatric Tummy Tuck
Scientific title	Postoperative intravenously administered iron sucrose 200 mg administered twice or postoperative orally administered iron for the treatment of post-bariatric abdominoplasty anaemia (ISAPA): a randomised controlled trial
Countries of recruitment	Brazil
Health conditions or problems studied	Postoperative anaemia, iron deficiency
Interventions	Study group: administration of 200 mg of iron sulphate intravenously after post-bariatric abdominoplasty, immediately after the operation and on the first postoperative day
	Control group: ferrous sulphate supplementation orally administered postoperatively at a dose of 1 tablet of 100 mg 12/12 h for 8 weeks.
Key inclusion and exclusion criteria	Inclusion criteria: women; 18 to 55 years old; previous bariatric surgery; body mass index (BMI) less than 32 kg/m^2^; stability of weight loss for at least 6 months
	Exclusion criteria: smoking; cholelithiasis demonstrated by ultrasound; uncontrolled systemic diseases; bone marrow diseases; haematological disorders; presence of renal or hepatic insufficiency; acute infection; prior use of intravenously administered iron in the last 3 months; haemoglobin level less than 11.5 g/dL; ferritin level less than 11 ng/mL or greater than 100 ng/mL; transferrin saturation index below 20 % or exceeding 50 %; vitamin B_12_ level less than 210 pg/mL; folic acid level less than 3.3 ng/mL; albumin level less than 3 g/dL; C-reactive protein greater than 5 mg/L; mean corpuscular volume less than 80 fL
Study type	Interventional
	Allocation: randomised
	Masking: unblinded
	Assignment: parallel
	Purpose: treatment
	Phase: not applicable
Date of first enrolment	April 2014
Target sample size	56
Recruitment status	Recruiting
Primary outcome	Haemoglobin level increase in the eighth week of postoperative abdominoplasty after bariatric surgery, measured by automated haematology counter in g/dL
Key secondary outcomes	Presence of iron deficiency assessed by serum ferritin, postoperative haemoglobin levels, fatigue measured by the Functional Assessment of Chronic Illness Therapy – Fatigue (FACIT-F) questionnaire, quality of life measured by the Short-Form 36 Health Survey (SF-36), adverse events, surgical complications. Timepoints: 1, 4 and 8 postoperative weeks

### Research ethics approval

This protocol was approved by the Research Ethics Committee of The Federal University of São Paulo (CEP UNIFESP) and is registered on the Brazil Platform under Presentation Certificate for Ethics Assessment (Certificado de Apresentação para Apreciação Ética – CAAE) number 11904213.3.1001.5505.

### Consent

The relevant information from this study will be provided to all patients in an understandable way. Patients will have the opportunity to have their questions answered and will provide their consent by signing the free and informed consent form.

### Methods: participants, interventions and outcomes

#### Study setting

The study is being conducted in Brazil, in the urban area of São Paulo city in two hospitals: Hospital São Paulo and Hospital do Servidor Público Estadual (State’s Civil Servants’ Hospital).

### Eligibility criteria

Patients who meet the inclusion and non-inclusion criteria, and provide their consent by signing an informed consent form, are eligible to the study.

### Inclusion criteria

Women, between 18 and 55 years of age, formerly obese patients treated with bariatric surgery using the vertical banded gastroplasty technique with Roux-en-Y gastric bypass by laparotomy, grade III deformity of the abdominal wall using the Pittsburgh rating scale [41], pre-bariatric BMI between 35 and 70 kg/m^2^ and post-bariatric BMI below 32 kg/m^2^ with stabilised weight loss for at least 6 months.

### Non-inclusion criteria

Illiteracy, tobacco use, cholelithiasis shown on ultrasound, uncontrolled systemic diseases, bone marrow diseases, haematological disorders, kidney or liver failure, acute infection, use of intravenously administered iron in the previous 3 months, prior anaphylactoid reaction with the use of intravenously administered iron, Hb levels less than 11.5 g/dL, ferritin levels less than 11 ng/mL or greater than 100 ng/mL, transferrin saturation index (TSI) greater than 50 % or less than 16 %, vitamin B_12_ levels less than 210 pg/mL, folic acid levels less than 3.3 ng/mL, albumin levels less than 3 g/dL, ultrasensitive C-reactive protein (CRP) levels greater than 5 mg/L, and mean corpuscular volume less than 80 fL.

### Exclusion criteria

The exclusion criteria include major intraoperative haemorrhaging with hemodynamic instability or haematoma formation requiring drainage in the operating room.

### Interventions

Patients in the study and control groups undergo a mixed-type abdominoplasty, also known as anchor-line abdominoplasty, based on a previously published procedure [42], under general anaesthesia, coordinated by the same surgeon for all the operations.

Both the study group and the control group are instructed to maintain, postoperatively, routine vitamin and mineral supplementation for post-bariatric patients, ensuring that the supplement Materna® (Wyeth Laboratory, Markham, ON, Canada), which contains 60 mg of iron, 1 mg of folic acid and 12 μg of vitamin B_12_, among other elements, is taken daily after breakfast.

Patients in the study group receive intravenously, during the immediate postoperative period, 200 mg of ferric hydroxide saccharate (Takeda Pharma Ltd., Berlin, Germany) diluted in 200 mL of saline over 60 minutes. The same dose is administered on the first postoperative day post-bariatric abdominoplasty, totalling 400 mg of intravenously administered iron supplementation for each patient in the study group. The iron is administered by the nursing team as specifically recommended for the administration of intravenous iron, with monitoring of vital signs and possible adverse reactions [43].

**Fig. 1:**
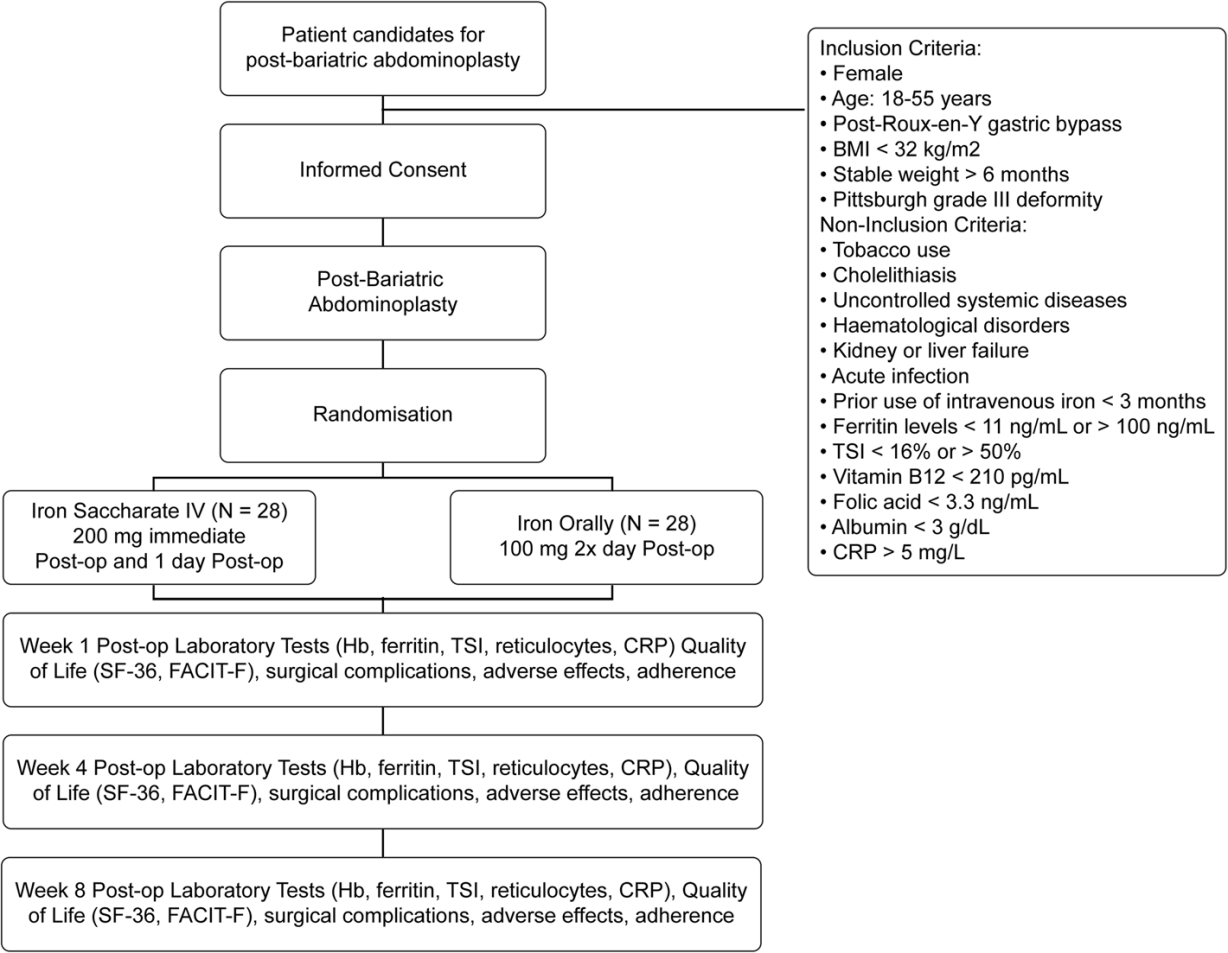
Trial flow diagram

Patients in the control group receive an additional postoperative orally administered supplementation of iron through the ingestion of one tablet of 330 mg of iron (III)-hydroxide polymaltose complex (equivalent to 100 mg of elemental iron) after lunch and another after dinner. Patients in the control group are instructed to maintain this additional orally administered iron supplementation during the 8-week follow-up period (Fig. 1).

### Interventions – modifications

#### Gastrointestinal upset

Iron polymaltose has a lower incidence of adverse effects than other ferrous salts [44]. However, patients are instructed to take the iron orally after meals to minimise gastrointestinal effects of the medication. Patients who report gastrointestinal symptoms may reduce the supplement dose to one tablet per day.

#### Allergic reactions

Allergic reactions to the use of intravenously administered iron are rare, and severe adverse effects have been reported only at doses above 400 mg [30]. In the case of an allergic reaction, the medication is suspended immediately, and the event is reported as an adverse event.

### Interventions – adherence

#### Adherence reminder sessions

Face-to-face reminder sessions as to the importance of treatment adherence occur the day before surgery and at each postoperative return appointment [45]. Such sessions include:

- The importance of following medical recommendations on the use of medications. In addition, to facilitate adherence, recommendations on the ingestion of medications are associated with the patients’ daily activities, specifically their meals
- Clear instructions, in writing and verbally, are provided for all prescriptions and, after asking whether the patient has any questions, the patient is asked to repeat the instructions in their own words

#### Adherence assessments

Two indirect methods to measure patient adherence are used: the Morisky-Green test (MGT) [46–48] and the Haynes-Sackett test [49, 50]. The MGT consists of four questions: (1) ‘Do you ever forget to take your medicine?’, (2) ‘Are you careless at times about taking your medicine?’, (3) ‘When you feel better, do you sometimes stop taking the medicine?’, (4) ‘Sometimes, if you feel worse when you take the medicine, do you stop taking it?’ An affirmative response to any one of these questions classifies the individual as non-adherent. The Haynes-Sackett test consists of the following statement and question: ‘Many people have some kind of problem with taking their medication. In the last 30 days, have you had difficulties with taking your medication?’ An affirmative response classifies the individual as non-adherent. Both methods are used during each patient’s return visit to quantify adherence to treatment.

### Interventions – concomitant care

All patients are discharged on the second postoperative day and provided with prescriptions of antibiotics (cefadroxil, 500 mg orally 12/12 h), analgesics (dipyrone, 1000 mg orally 6/6 h) and anti-inflammatory drugs (celecoxib, 200 mg orally 12/12 h) for use during the first postoperative week. Suction drains are removed when drainage is less than 50 mL in 24 hours. A restrictive practice was adopted for blood transfusions, in which none of the patients receive blood transfusion while their Hb levels remain above 7 g/dL [51].

Patients receive a list with nutritional guidelines that advise them to eat six times per day, chew their food well, avoid fatty foods and sweets and include fruits and vegetables in their diet. The use of other food or vitamin supplements is not permitted other than those recommended by the study during follow-up.

### Outcomes

#### Primary outcome measures

The primary outcome is the detection of a minimum difference of 1.5 g/dL in the final mean values of Hb levels at the eighth postoperative week between the study and control groups.

### Secondary outcome measures

#### Laboratory variables

In addition to Hb levels, haematocrit levels, ferritin levels, the TSI, the reticulocyte count and CRP levels are measured preoperatively, the day before surgery and at one, four and eight postoperative weeks. At the end of follow-up (8 weeks), the proportions of patients with anaemia (Hb below 12 g/dL) and without anaemia (Hb above 12 g/dL) and with iron deficiency (ferritin below 30 ng/mL) and without iron deficiency (ferritin above 30 ng/mL) are evaluated, together with the percentages of success in the full recovery of preoperative Hb levels among the patients in the study and control groups. Laboratory tests are conducted in the Hospital do Servidor Estadual and Hospital São Paulo laboratories, following the recommendations of the Brazilian Society of Clinical Pathology and Laboratory Medicine (Sociedade Brasileira de Patologia Clínica e Medicina Laboratorial – SBPC/ML). All instruments used in this study were subjected to internal (the manufacturer) and external controls through the Program of Excellence for Medical Laboratories (Programa de Excelência para Laboratórios Médicos) associated with SBPC/ML.

#### Quality of life

Quality of life is assessed using a generic questionnaire (Short-Form 36 Health Survey (SF-36)) [52] and a specific questionnaire to assess fatigue and other anaemia-related symptoms (Functional Assessment of Chronic Illness Therapy – Fatigue (FACIT-F)) [53], both translated and validated for Portuguese, applied using an assisted self-administered questionnaire the day before surgery and then at one, four and eight postoperative weeks.

#### Adverse effects

Adverse effects will be estimated by measuring the differences between the two groups in regards to the proportions of mild (grades 1 and 2) and severe (grade 3, 4 and 5) adverse effects, which are possibly, probably, or definitely related to the use of iron supplementation during the follow-up period. Version 4 of Common Terminology Criteria for Adverse Events (CTAE v4.0) [54] will be used to grade adverse effects. The following adverse effects will be considered mild: transitory development of a skin rash, itching, tachycardia, tremor, dyspepsia, constipation, and nausea with improvement after discontinuation of medication or of symptomatic treatment. The following adverse effects will be considered severe: the maintenance of symptoms even after discontinuing the medication and treatment, need for hospitalisation, bronchospasm, anaphylactic shock and death. At each visit, patients will be asked about adverse events that have manifested since their previous visit. A standardised list containing the most common adverse effects of iron supplementation will be used. Cases where there is a need to definitively discontinue use of the medication will be considered treatment failures.

#### Surgical complications

Postoperative complications, divided into systemic and local, are evaluated over the 8 weeks of postoperative follow-up, and their percentages are compared between the study and control groups. The following complications are considered systemic: the need for hospital readmission, postoperative blood loss with an indication for blood transfusion, deep vein thrombosis (diagnosed by ultrasound examination), pulmonary embolism (diagnosed using an angiographic examination) and death.

The following complications are considered local: seroma (accumulation of serous subcutaneous fluid evident upon physical examination after removing the drain and following at least one aspiration); haematoma (accumulation of haematic subcutaneous fluid evident upon physical examination after removing the drain and following at least one aspiration); wound infection (based on the definition established by the Centers for Disease Control and Prevention (CDC) [55]); wound dehiscence (defined as separation of the wound margins requiring medical or surgical treatment [56]); and necrosis (defined by the presence of devitalised tissue that extends through the full thickness of the skin, requiring surgical debridement).

#### Dietary survey

To determine whether there are differences in the iron dietary intake levels between the two groups, a dietary survey is conducted. At the eighth postoperative week, patients answer a 24-hour diet recall survey in which they report all food and drinks consumed during the previous day, from breakfast to dinner [57]. The recall is administered by a professional dietician following the recommendations of the Nordic Cooperation Group of Dietary Researchers [58]. NutWin version 3.0 software, developed and validated by the Federal University of São Paulo (Universidade Federal de São Paulo), is used to calculate dietary iron intake [59].

#### Menstruation

Because prolonged menstrual bleeding lasting more than 7 days can have an impact on Hb levels [60], all patients are asked during the follow-up period whether they have had menstrual bleeding lasting for more than 7 days.

### Sample size

A sample size of 56 patients (28 per group) was calculated, assuming an 80 % statistical power to detect a minimum difference in Hb levels of 1.5 g/dL (the primary outcome), considering a standard deviation for Hb levels of 1.84 [61], a two-tailed significance level of 0.05 and a patient loss to follow-up of 10 %.

The need for postoperative follow-up assessments of participants, the relatively brief 8-week follow-up period, the flexibility to decrease the iron oral dose to one tablet daily for patients reporting gastrointestinal symptoms, and reminder sessions for treatment adherence might reduce participant attrition and, consequently, the attrition bias.

### Recruitment

Patients are recruited from the post-bariatric plastic surgery outpatient clinics of Hospital do Servidor Estadual and Hospital São Paulo via local advertisements. Patients on hospital waiting lists are also identified. Both hospitals have active bariatric surgery services that regularly refer patients to the post-bariatric plastic surgery outpatient clinic; therefore, we do not expect challenges regarding patient recruitment.

### Methods – assignment of interventions

#### Allocation – sequence generation

The patients are allocated to the study or control group at a ratio of 1:1 using a computer-generated randomiser (http://www.randomization.com) with fixed block sizes. The block sizes are kept confidential.

#### Allocation – concealment mechanism

Opaque, sealed, serially numbered envelopes containing carbon paper and aluminium foil inside are used to maintain the confidentiality of the random allocation. The researchers responsible for the recruitment do not receive any information regarding the groups into which the patients are allocated. The allocation of the study participants was defined using a computer-generated random sequence. Confidentiality of the block size and blinding patient allocation using a series of opaque, sealed, and numbered envelopes were used to reduce selection bias.

#### Allocation: implementation

A surgical team member who did not participate in the recruitment of patients generated the allocation sequence and maintained the allocation concealment. The researchers responsible for recruitment did not receive any information regarding the patient allocation groups. The sequentially numbered envelopes remained stored with the surgical team member throughout the study. Prior to the post-bariatric abdominoplasty surgery, the surgeon wrote the name of the patient on the envelope that corresponded to the numerical sequence of the research. At the end of the surgery, the envelope was opened, revealing the research group to which the patient belonged.

### Blinding

Due to the nature of the intervention (orally administered iron supplements result in blackening of the stool [62], facilitating the identification of the allocation group), we chose not to blind the patients or researchers to the allocation group. However, the surgeon who evaluates the incidence of local complications is blinded to the allocation group. In addition, the patients are not informed about the outcome of their laboratory tests to prevent the knowledge of their test results from influencing their responses on the quality of life questionnaires [63]. The use of an objective laboratory measurement as the primary endpoint (Hb level) also reduces detection bias.

Although unmasked clinical trials are subject to performance bias, the disclosure of allocation concealment at the end of the surgery reduces the possibility of surgical team performance bias during surgery. In addition, both the recommendation to use the same vitamin and mineral supplementation and the written supply of the same nutritional guidelines for all participants aim to reduce performance bias over the postoperative follow-up period.

### Methods – data collection, management, and analysis

#### Data collection methods

Researchers collect patient demographic data using a standardised form for all study patients.

#### Data collection methods – retention

The night before each outpatient visit, patients are contacted by telephone and reminded of the importance of collecting blood for laboratory tests, completing the quality of life questionnaires, and the medical evaluation.

#### Data management

All data collected during the study are entered into two separate spreadsheets at different times. The two spreadsheets are then compared with each other to ensure the accuracy of the data collected [64].

### Statistical methods

#### Statistical methods – outcomes

The data will be entered into the statistical software Statistical Package for the Social Sciences (SPSS), version 17.0, and initially evaluated descriptively using summary measures (means, standard deviations, minima, maxima, and quartiles). To guarantee reliability and avoid selective outcome reporting, a fully specified statistical analysis plan will be written before unmasking.

To analyse the behaviour of variables for quality of life, Hb level, reticulocyte count, iron profile and CRP level at different times, a repeated measure analysis of variance (ANOVA) will be employed. This model will presuppose the normality of the data tested in the residuals (difference between the observed and estimated value) of the fitted model using the Kolmogorov-Smirnov test.

To determine the presence of an interaction between group and time, the means of the variables between groups will be compared at each time-point using Student’s *t* test for independent samples, and means between different time-points for each group will be compared using Student’s *t* test for paired samples.

For those variables in which there is a lack of normality in the data, non-parametric Mann-Whitney tests will be performed to compare the differences between groups at each time-point, and the Wilcoxon test will be used to compare patterns over time for each group. Bonferroni correction will be used for these tests to ensure an overall significance level of 5 %.

### Statistical methods: additional analyses

#### Adjusted analyses

The final mean Hb levels in the eighth postoperative week (primary outcome) will be adjusted for baseline Hb level, ferritin levels, and TSI via an analysis of covariance (ANCOVA). Both the adjusted and unadjusted analyses will be reported in the final publication.

### Statistical methods – analysis population and missing data

Data analysis will be performed based on the original allocation of all patients as defined by randomisation, regardless of the degree of adherence to the protocol (intention-to-treat principle).

With respect to missing data, the amount thereof, their patterns and variables associated with omission will define the most appropriate technique to be used in the processing of such data, whether in the primary analysis or the sensitivity analysis. For primary analyses, methods will be used that make use of all available data, such as multiple imputations. To reduce the presence of incomplete data and attrition bias, the need to discontinue the allocated treatment will be considered as treatment failure.

### Dissemination

#### Protocol amendments

Any changes to the protocol that might affect the study, the potential benefits for patients, or their safety (including changes to the study objectives, design, patient population, sample size, study procedures, or significant administrative aspects) will require a formal amendment to the protocol. These changes will be agreed upon among the researchers and will require approval from the Research Ethics Committee before implementation.

### Confidentiality

All participant information will be kept in password-protected files, and the identifiable data will be replaced by codes.

### Access to data

All of the researchers involved in this study will have access to all of the collected data.

### Dissemination policy – authorship

The criteria for authorship of this protocol followed the guidelines established by the International Committee of Medical Journal Editors [65]. A team of professional translators was used to translate the text from Portuguese into English. The final report will follow the Consolidated Standards of Reporting Trials (CONSORT) 2010 guidelines.

## Discussion

Anaemia is the second most frequent complication following post-bariatric abdominoplasty [7]. Two months after surgery, these patients have still not recovered their preoperative Hb levels, and 45 % develop severe iron deficiency [13]. However, the recovery of Hb levels is considered an important factor in postoperative patient recovery [23]. This aspect becomes even more relevant in post-bariatric patients, as they often undergo a series of subsequent surgical procedures to treat deformities in different regions of the body; thus, they only have a short time to recover their haematological parameters [24].

A systematic review concluded that iron supplementation is effective in promoting an increase in Hb levels [37]. Although iron supplementation can be administered either orally or intravenously, it has been hypothesised that intravenous administration has improved efficacy in the population of post-bariatric patients given the difficulties with orally administered iron absorption in these patients [12]. This randomised clinical trial protocol was designed to test this hypothesis because no studies were found in the literature that evaluated the effect of iron supplementation in a population of patients undergoing post-bariatric surgery.

Blinding is commonly used in randomised clinical trials to reduce the risk of bias [66]. However, because orally administered iron supplementation can cause stool colour changes, the implementation of blinding in this study was considered to be impractical. In fact, most published randomised clinical trials evaluating the effect of iron supplementation in postpartum women were not blinded [67].

The primary outcome of this study is the detection of a minimum difference of 1.5 g/dL in the final mean values of Hb levels at the eighth postoperative week between the study and control groups. This difference in Hb levels was established based on the fact that the patients in the study group receive a total of 400 mg of intravenously administered iron, and 200 mg of iron is needed to increase Hb levels by approximately 1 g/dL [31]. This dose would be sufficient to recover the loss of approximately 2.1 g/dL of Hb that occurs during this type of surgery [13]. Because the control patients receive iron orally and the absorption of iron in these patients is compromised as a result of bariatric surgery [38], a difference in Hb levels of 1.5 g/dL between the study and control groups was considered to be biologically plausible.

The choice of a laboratory variable as a primary outcome rather than a more relevant clinical outcome, such as reduced blood transfusion rate or improved quality of life, could be considered a limitation of this study [68]. However, because each 1 g/dL increase in Hb increases the SF-36 vitality score from 1.2 to 7.4 points [69] and the minimum clinically relevant difference in the SF-36 vitality score is 5 points [70], a difference of 1.5 g/dL in Hb levels was considered to have the potential to be clinically relevant. Consequently, the response of Hb levels to treatment was defined as the primary outcome, as published in other protocols [71]. The eighth postoperative week was determined as the primary time-point for haemoglobinometric measurements because the postoperative recovery of Hb levels largely occurs between 4 and 6 weeks following major surgery [72, 73].

It is unlikely that the use of intravenously administered iron compared to orally administered iron can significantly alter relevant clinical outcomes, such as hospital readmission or mortality rates. However, there is an increasing recognition of the importance of the benefits of small interventions in surgical care as opposed to a single ‘magical’ intervention to improve outcomes following surgery [74]. Furthermore, iron supplementation runs counter to a paradigm shift known as patient blood management.

Patient blood management has been increasingly accepted as an effective means of reducing blood transfusion rates and improving postoperative outcomes [75]. It is defined as the application of evidence-based medical and surgical concepts aimed at superior clinical outcomes through the improved utilisation of the patient’s own blood instead of donor blood. Such management is intended to maintain the concentration of Hb, to optimise homeostasis and to minimise blood loss. It consists of three pillars: (1) optimising erythropoiesis, (2) minimising blood loss, and (3) promoting a greater tolerance to anaemia [76]. Intravenously administered iron supplementation falls under the first pillar. The proposed study represents a randomised clinical trial designed to assess the effect of intravenously administered iron supplementation in treating anaemia following post-bariatric abdominoplasty.

### Trial status

Recruiting. The first patient was randomised in April 2014; by August 2015, 19 patients had undergone surgery.

## Abbreviations

FACIT-F: Functional Assessment of Chronic Illness Therapy – Fatigue
BMI: body mass index
Hb: haemoglobin
TSI: transferrin saturation index
CRP: C-reactive protein
SF-36: Short-Form 36 Health Survey
CTAE: Common Terminology Criteria for Adverse Events
CDC: Centers for Disease Control
USA: United States of America
CAAE: Certificado de Apresentação para Apreciação Ética (Presentation Certificate for Ethical Assessment)
